# Conflicts in Gaza and around the world create a perfect storm for infectious disease outbreaks

**DOI:** 10.1371/journal.pgph.0002927

**Published:** 2024-02-07

**Authors:** John E. Kearney, Natalie Thiel, Arian El-Taher, Sabreen Akhter, David A. Townes, Indi Trehan, Paul S. Pottinger

**Affiliations:** 1 University of Washington School of Medicine, University of Washington, Seattle, Washington, United States of America; 2 Department of Pediatrics, University of Washington School of Medicine, University of Washington, Seattle, Washington, United States of America; 3 Department of Global Health, University of Washington School of Medicine, University of Washington, Seattle, Washington, United States of America; 4 Department of Emergency Medicine, University of Washington School of Medicine, University of Washington, Seattle, Washington, United States of America; 5 Department of Epidemiology, University of Washington School of Medicine, University of Washington, Seattle, Washington, United States of America; 6 Department of Medicine, University of Washington School of Medicine, University of Washington, Seattle, Washington, United States of America; PLOS: Public Library of Science, UNITED STATES; McGill University, CANADA

The global history of conflicts has revealed that infectious diseases resulting from displacement, overcrowding, healthcare collapse, destruction of critical water and sanitation infrastructure, and malnutrition can cause death rates equal or beyond those from direct violence [1]. The crisis in Gaza is no exception and has created a perfect storm for infectious disease outbreaks.

## The crisis in Gaza

As of January 24, 2024, an estimated 25,700 Palestinians have been killed—approximately 70% of whom are women and children—and at least 68,000 civilians injured in Gaza [2, 3]. Delivery of aid to Gaza is severely restricted due to a longstanding blockade that has tightened in the last three months [2]. Two-million out of 2.2 million people are internally displaced. Israel’s bombing campaign has destroyed hospitals, killed over 300 healthcare workers, and leveled infrastructure [4]. Attacks have been recorded on the largest medical facilities in Gaza resulting in an unprecedented collapse of the healthcare sector in Gaza, with only 14 of 36 hospitals across Gaza still partially operational [2]. There is limited supply of potable water and sanitation is severely compromised.

The World Health Organization (WHO) warned about the high risk of infectious diseases in shelters for internally displaced persons (IDP) in Gaza [5]. Due to severe overcrowding in shelters and the disruption of health and sanitation systems, there are already reports of infectious disease outbreaks at IDP shelters, including infectious diarrhea, dysentery, respiratory infections, skin infections, and hepatitis A [2, 6]. While accurate case numbers are unknown due to insufficient surveillance, healthcare workers report unsanitary conditions as well as outbreaks of jaundice and diarrhea [6]. The WHO noted outbreaks of acute respiratory infections, scabies, lice, diarrhea, skin rash, chickenpox, and hepatitis associated jaundice [7]. Over half of the cases of diarrhea are in children under age five, a rate 23 times higher than in 2022 [7].

Reports of infections in Israeli soldiers returning from conflict in Gaza can provide a lens into the infectious disease threats in the region. Several Israeli soldiers have died after contracting multidrug resistant infections [4]. The Israeli Association for Infectious Diseases reports highly resistant strains of *Klebsiella*, *Escherichia coli*, and *Aspergillus* in returning Israeli soldiers. Due to the inability to consistently ensure standardized infection prevention measures, the threat of drug-resistant bacterial infections has always loomed over Gaza and the current conflict poses significant risk of creating further challenges to antimicrobial resistance. For example, a reconstructive surgical unit in Gaza had found 70% of positive bacterial cultures from patients were multidrug resistant in November 2023 [8]. In 2022, MSF (Médecins Sans Frontières or Doctors Without Borders) monitoring projects had found the majority of *Staphylococcus aureus* isolates were methicillin resistant and over 30% of Gram-negative isolates were multidrug resistant with extended-spectrum beta-lactamases. These trends that predate the current crisis can only be expected to get worse now. With 52,000 pregnant women in Gaza and an estimated 183 births per day, there is also a greater risk of obstetric complications including peripartum, maternal, and neonatal infection and sepsis [7].

## Infectious diseases in conflict zones

History has shown how conflict creates environments that favor the spread of infectious diseases. Conflicts in Iraq, Syria, and Yemen have seen the destruction of healthcare infrastructure, contamination of water sources, and disruption of food supplies, leading to outbreaks of cholera, poliomyelitis, measles, cutaneous leishmaniasis, and diphtheria [9]. There are long-term consequences of conflict due to the disruption of routine vaccination programs as well as disease surveillance and response systems. Historically, this has manifested as a resurgence in preventable outbreaks such as malaria in Tajikistan, yellow fever throughout parts of Africa, and poliomyelitis in Somalia, Sudan, Pakistan, and Afghanistan [10–12]. Syria successfully eliminated measles in 1999 but experienced more than 30,000 cases after the start of the Syrian conflict in 2015 due to interruption of vaccination, lack of healthcare access, and overcrowding from mass internal displacement [13]. In Yemen, cholera increased dramatically after the start of their civil war in 2014 and is now the largest cholera outbreak in modern history [14].

For children, crowded living conditions, delays in routine immunizations, and malnutrition are associated with numerous infectious outbreaks [9]. Deaths in children under age five from measles can nearly double in populations experiencing conflict [15]. Outbreaks of measles in Asia and Africa in mostly post-conflict settings were the result of poor vaccination status, leading to case fatality rates exceeding 5%. During prolonged conflicts, poverty and overcrowding results in “diseases of exploitation,” resulting in transmission of HIV and other STIs. Poor hygiene facilitates the transmission of louse-borne diseases like epidemic typhus, trench fever, and relapsing fever [16].

Malnutrition is a known contributor to the childhood burden of infectious disease in low- and middle-income countries. There is a clear relationship between malnutrition and outbreaks of tuberculosis [17]. Malnutrition is an underlying cause in 40–60% of deaths attributable to diarrhea, pneumonia, and measles [16]. Infection can then worsen malnutrition through decreased intake, malabsorption, and increased metabolic needs [16]. Even before the current conflict, 63% of Gazans experienced food insecurity; in food insecure households, 30.4% of children under five were underweight, 32.8% stunted, and 9.6% wasted [18]. Now, it is estimated that 2.2 million Palestinians are at imminent risk of famine, with almost 400,000 currently facing extreme lack of food and starvation [2].

## Critical actions in Gaza and other conflict areas

On November 27, 2023, the UN called for “a full humanitarian ceasefire, for the benefit of the people of Gaza, Israel and the wider region” [19]. On November 10 and again on December 16, the WHO called for a ceasefire. The medical community must join this growing call and advocate for an immediate and permanent ceasefire to treat current infectious disease outbreaks and shore up the health system to prevent future ones.

After the cessation of hostilities, relief efforts must be coordinated closely with the Gaza Ministry of Health to ensure that aid is effectively distributed and addresses the most urgent needs. Frameworks such as the Sphere standards, which provide detailed guidance on humanitarian response within a rights-based framework, can inform this work [20]. Relief efforts should also collaborate closely with public health surveillance systems such as the WHO Early Warning, Alert and Response Network (EWARNS) to ensure timely and accurate data on illnesses that can direct outbreak response and prevention. Moreover, there must be secure and unimpeded access to Gaza for humanitarian aid delivery.

Epidemiological surveillance of diarrheal and respiratory outbreaks could be aided by access to rapid multiplex molecular testing kits. First-line and advanced antimicrobials must be made readily available. Fundamental infection control techniques can mitigate the spread of antimicrobial-resistant infections. Some of these immediate medical needs could be filled by WHO Interagency Emergency Health Kits, which are preformulated sets of drugs, supplies, and equipment. These kits are also available for specific diseases with outbreak potential, such as measles and cholera [21]. Repairing and upgrading sanitation systems will be essential in preventing waterborne diseases. Reactivation of the water desalination plant and water wells throughout Gaza will help address this need [2]. Lastly, a primary challenge that must be addressed is providing adequate nutrition to avert the risk of famine.

These interventions are applicable in all conflict areas. It is imperative that the global community, particularly the infectious diseases community, take action to mitigate any further morbidity and mortality in Gaza and all humanitarian crises.
